# Gender dysphoria in a child with 46, XX DSD due to salt wasting CAH at a tertiary care setting: A case report

**DOI:** 10.1192/j.eurpsy.2026.11139

**Published:** 2026-07-31

**Authors:** D. Munipati, R. Sagar, V. Jain, S. Priyadarshini

**Affiliations:** 1 Psychiatry; 2 Pediatrics; 3 Pediatrics Endocrinology, AIIMS, New Delhi, India

## Abstract

**Introduction:**

K was born at term via spontaneous vaginal delivery (uneventful perinatal course). The baby had atypical genitalia and was assigned male gender at birth. He was brought to our Institute (Department of Pediatrics) at 1 month with poor feeding, poor weight gain, progressive hyperpigmentation, and recurrent episodes of vomiting. Based on Prader stage 3 genitalia, hyponatremia, hyperkalemia, metabolic acidosis, elevated 17 hydroxyprogesterone, presence of a uterus on sonogram, and 46, XX karyotype; a diagnosis of salt wasting CAH was made. Parents counselled that the most appropriate gender of rearing for the baby would be female. Subsequently, K was reared as a female, later lost to follow-up. Upon returning at 6 years, K exhibited severe virilization, including increased phallic length and male gender role behaviour. On Psychiatry evaluation, K expressed distress to take up assigned gender (female) role and affinity to opposite gender role. K didn’t attend school or step out of his house for fear of being bullied, would have significant anger outbursts. Over time, poor compliance to medical management with glucocorticoids led to continued high endogenous androgen production, contributing to Gender dysphoria. A multidisciplinary approach was taken, K was started on low dose SSRI to help with the dysphoria and was discussed to continue on going gender affirming medical management.

**Objectives:**

To highlight the various mental health issues faced by a child diagnosed with 46 XX DSD, salt wasting CAH.

**Methods:**

Pateint first presented to us at age of 1 month of age and later after medical stabilization lost the patient to follow-up. Later after 6 years when child was 6 years of age was bought back after which regular follow-up at 3months interval revealed ongoing multiple behavioral issues and school refusal.

**Results:**

This child was diagnosed with Gender dysphoria, Oppositional Defiant disorder and required medication to alleviate distress associated with both.

**Conclusions:**

It is important to assess for mental health issues in children with Differences of sexual development.

**Disclosure of Interest:**

None Declared

